# Determination of the Volatile Composition in Essential Oil of *Descurainia sophia* (L.) Webb ex Prantl (Flixweed) by Gas Chromatography/Mass Spectrometry(GC/MS)

**DOI:** 10.3390/molecules15010233

**Published:** 2010-01-08

**Authors:** Jing Li, Xingang Liu, Fengshou Dong, Jun Xu, Yongquan Zheng, Weili Shan

**Affiliations:** 1 Key Laboratory of Pesticide Chemistry and Application, Institute of Plant Protection, Chinese Academy of Agricultural Sciences, Ministry of Agriculture, Beijing, 100193, China; E-Mail: Lijing2011@gmail.com (J.L.); 2 Institute for Control of Agrochemicals, Ministry of Agriculture, Beijing, 100125, China; E-Mail: shanweili@agri.gov.cn (W.S.)

**Keywords:** *Descurainia sophia* (L.) Webb ex Prantl, essential oil, GC/MS, *cis*-β-ocimene, menthol

## Abstract

Exhaustive hydro-distillation of *Descurainia* *sophia* (L.) Webb ex Prantl (flixweed) collected from two different locations (Cangzhou city-Sample 1 and Beijing city-Sample 2) gave in both cases yellowish colored oils in 0.31 and 0.26% yield, respectively. Detailed chemical composition of the essential oils was analyzed by GC and GC/MS, and forty and thirty-eight compounds were identified. The results indicated that the most abundant component of Sample 1 were *cis*-β-ocimene (20.1%), menthol (11.27%), neoisomenthyl acetate (3.5%), alloaromadendrene (2.28%) and longicyclene (2.25%). Compared with the constituents of Sample 1, several chemical compounds such as 1,8-cineole, α-eudesmol, *cis, trans*-farnesol and β-pinene were not detected in Sample 2 although it was similarly dominated by *cis*-β-ocimene (17.12%), menthol (10.7%) and neoisomenthyl acetate (2.96%). Final analysis of the chemical constituents in the essential oils of the two samples showed similarity in their chemical composition, but the relative content of all shared chemical constituents in Sample 2 was lower than that in Sample 1.

## Introduction

*Descurainia sophia* (L.) Webb ex Prantl (flixweed), which originated in South Europe and North Africa, is a member of family Brassicaceae and one of the most troublesome annual dicot weeds which is widely distributed throughout most of the major wheat producing regions in China [1,2,3]. It is an early seral species able to quickly invade cultivated wheat fields and is quite competitive for moisture and nutrients, reducing overall wheat yields [4,5]. It has also been widely used in folk medicine. In the Middle Asia, the decoction of the aerial part is used for throat diseases and as an antipyretic for measles and smallpox. The tincture is used as a diuretic, antihelmintic, and hemostatic for internal hemorrhages [6]. Seeds of flixweed have also been used in China as a Chinese Traditional Medicine (TCM) to relieve cough, prevent asthma, reduce edema, promote urination and for their cardiotonic effect. In some cases, the seeds can also be used in the treatment of some cancers [7].

As medicinal materials, this plant is very important for its chemical composition. Up to now, several phytochemical studies have identified the presence of cardiac glycosides and glucosinolate degradation products such as isothiocyanates, nitriles, and epithiobutane derivates in its seeds and dried aerial parts of the plant [8], and some new lactones and aryldihydronaphthoic acid in the seeds were also reported [9,10,11,12,13]. However, the chemical composition of volatile compounds of flixweed has not been investigated yet. 

Moreover, previous studies reported that the chemical composition of the essential oil derived from the plant can be influenced by many factors, such as genetic structure, climatic factors and agronomical practices. In order to make a thorough investigation into the chemical constituent of the essential oil of *Descurainia sophia*, it is important to evaluate the influence of different ecological locations on the content and composition of the essential oil. The aim of this work was to evaluate and compare the content and composition of the essential oil between two samples of *Descurainia sophia* collected from different geographic regions (Cangzhou city and Beijing city) by using the hydrodistillation and GC-MS techniques.

## Results and Discussion

The essential oils obtained by steam distillation of aerial parts of *Descurainia sophia* collected from the two different geographical regions were isolated with a yield of 0.31 and 0.26% (based on dried plant material), respectively. The isolated oils were both yellowish liquids with a strong aromatic fragrance.

The results of the GC and GC/MS analysis of two samples of *Descurainia sophia* are presented in Table 1. A total of 42 volatile compounds were identified in the two samples. It is apparent from the data that the two samples were abundant in monoterpenes, sesquiterpenes and their derivatives. 

**Table 1:** Chemical composition of two samples of *Descurainia sophia* (Flixweed) and their corresponding retention indices (RI).

Peak Number	Compound	Experimentally determined Kováts Indexes	Relative amount (%)^c^	
			Cangzhou (S1)	Beijing (S2)
1	tricyclene *^a,b^*	921	0.2	0.17
2	α-pinene*^ a,b^*	940	0.47	0.35
3	camphene *^a,b^*	948	0.35	0.11
4	sabinene *^a.b^*	972	0.17	0.13
5	β-pinene *^a.b^*	983	0.16	-^d^
6	myrcene *^b^*	990	0.38	0.34
7	α-terpinene *^a,b^*	1015	0.63	0.53
8	1,8-cineole*^ a,b^*	1021	2.58	-
9	*cis*-β-ocimene*^ a,b ^*	1034	20.1	17.12
10	*trans*-β-ocimene*^ a,b^*	1045	1.07	0.81
11	terpinolene*^ b^*	1083	0.54	0.26
12	α-pinene oxide*^ a,b^*	1103	0.33	0.26
13	*trans*-allo-ocimene*^ a.b^*	1115	0.96	0.69
14	*trans*-tagetone*^ a,b^*	1124	1.04	0.71
15	*cis*-allo-ocimene*^ a.b^*	1130	-	0.24
16	isoborneol*^ a,b^*	1142	0.37	0.31
17	menthol*^ a,b^*	1162	11.27	10.7
18	isomenthol*^ a,b^*	1176	0.27	0.23
19	neoisomenthol*^ a,b^*	1183	1.77	1.29
20	nerol*^ a,b^*	1215	1.33	1.33
21	thymol*^ a,b^*	1260	0.99	0.71
22	bornyl acetate*^ a,b^*	1280	0.81	0.7
23	neoisomenthyl acetate*^ a,b^*	1298	3.5	2.96
24	terpinyl acetate*^ a,b^*	1330	1.88	1.31
25	α-longipinene*^ a,b^*	1352	1.41	1.1
26	longicyclene*^ a,b^*	1374	2.25	1.69
27	cyperene*^ a,b^*	1393	1.76	1.34
28	*trans*-β-farnesene*^ a,b^*	1428	-	0.27
29	*trans,trans*-farnesol*^ a,b^*	1450	0.72	0.48
30	alloaromadendrene*^ a,b^*	1475	2.28	2.18
31	α-bisabolene*^ a,b^*	1502	1.61	1.06
32	β-curcumene*^ a,b^*	1509	0.82	0.61
33	α-selinene*^ a,b^*	1521	0.7	0.43
34	*cis*-nerolidol*^ a,b^*	1539	0.76	0.54
35	longipinanol*^ a,b^*	1559	0.54	0.38
36	spathulenol*^ a,b^*	1580	0.18	0.15
37	cedrol*^ a,b^*	1610	1.43	0.88
38	β-cedren-9-α-ol*^ a,b^*	1649	0.79	0.63
39	α-eudesmol*^ a,b^*	1662	0.57	-
40	α-santalol*^ a,b^*	1677	1.18	1.04
41	*cis, trans*-farnesol*^ a,b^*	1701	0.53	-
42	α-fenchene*^ a,b^*	1772	1.48	1.16
	identified monoterpenes and derivatives		52.35	42.42
	identified sesquiterpenes and derivatives		17.08	12.78

Compounds are listed in order of elution. Retention indices were calculated from retention times relative to those of *n*-alkanes (C_6_ to C_28_) on the non-polar AB-5 column; ^a^ Identification by comparison of retention indices to those of authentic samples; ^b^ Identification based on comparison of mass spectra with NIST 05 library database; ^c^ Percentages obtained by GC-FID peak-area normalization; ^d^ not detected.

Fifty-four components were detected in Sample 1(S1), of which forty components, representing 69.43% of the total volatiles, were identified by comparison of their retention indexes and the mass spectra of each GC component with those of standards and with reported data. According to our results, the major constituents were *cis*-β-ocimene (20.1%), menthol (11.27%), neoisomenthyl acetate (3.5%), 1,8-cineole(2.58%), alloaromadendrene (2.28%), longicyclene (2.25%), terpinyl acetate (1.88%), neoisomenthol (1.77%), cyperene (1.76%) and α-bisabolene (1.61%). These components account for 49% of the total volatile composition, while the other minor components make up the balance. The remaining components of Sample 1 were tentatively identified to be esters (7.57%), aldehydes (4.67%), alcohols (3.08%), phenols (2.95%) and ketones (3.52%) by comparison of their recorded mass spectra with data from the NIST 05 mass library only.

Out of forty-eight peaks detected in Sample 2 (S2), thirty-eight components, which accounted for 55.20%, were identified in the volatile oil. The oil derived from S2 is dominated by *cis*-β-ocimene (17.12%), menthol (10.70%), neoisomenthyl acetate (2.96%) and alloaromadendrene (2.28%). Based on the preliminary analysis of the remaining components of Sample 2 (amounting to 44.8% of the total volatiles) by comparison of mass spectra with the MS library, several chemical compound classes such as esters (9.38%), aldehydes (5.38%), phenols (3.28%), fatty acids (3.32%) and ketones (6.55%) were tentatively identified. Our analysis of the chemical constituents in the essential oil of the two samples of *Descurainia sophia* showed similarity in their chemical composition. Thirty six compounds, which accounted for 66.34% and 54.69% of the total volatiles, respectively, were common to the two samples, and *cis*-β-ocimene, menthol, neoisomenthyl acetate were identified as the main constituents in both of them. Monoterpenes and sesquiterpenes dominated in the two samples, separately accounting for (52.35% and 17.08% in Sample 1, respectively) and (42.42% and 12.78% in Sample 2, respectively) of the total. The percentages of the peak area relative to the total peak area of the identified compounds were also given in Table 1, and these data demonstrate the composition was different not only quantitatively but also qualitatively. For instance, 1,8-cineole and α-eudesmol, which accounted for 2.58% and 0.57% of the volatile oil of S1, respectively, were not identified in S2. Two of the identified chemical compounds in sample B such as *cis*-allo-ocimene and *trans,trans*-farnesol, which accounted for 0.24% and 0.27%, respectively, were not detected in Sample 1.

A large amount of literature has reported that chemical composition of volatile oils could differ widely within the same species across different geographical locations where the contrast of the biotic and abiotic factors such as ecological niches, climatic conditions and trophic level is very sharp [14,15,16,17]. Telci *et al*. [14] verified that the compositions of the essential oils from two varieties of *Coriandrum sativum* growing in two different locations were qualitatively similar, but variation in the oil content between varieties was significant [14]. In addition, the yields and composition of the essential oils extracted from fruits of coriander (*Coriandrum sativum* L.) growing in two different Tunisian regions were reported in literature [15], it is apparent from the results that the essential oil yields and composition varied significantly among the different growing locations. Kokkini *et al.* [16] studied the essential oils from *Origanum vulgare* subsp*. hirtum* plants collected in late autumn from six localities of three distinct geographic areas of Greece. They reported that oils of plants from the Northern part of Greece were rich in thymol, whereas those from the Southern part of the country were rich in carvacrol.

However, from the data of the chemical constituents in the volatile oil of the two samples of *Descurainia sophia* in our manuscript, it is obvious that there is a great similarity in their chemical composition, but the relative amount of all the thirty six shared chemical constituents such as *cis*-β-ocimene and menthol in sample 1 collected from Cangzhou city were higher than that in sample 2, which was collected from Beijing city. Comparing the present data in our manuscript with those previously reported in the literature on the essential oil compositions of a plant from different geographical regions, our results also apparently showed that different growing regions had a significant effect on the content of essential oil of *Descurainia sophia*. And the observed differences of the content of essential oil derived from the two samples might be caused by the different ecological environment, climatic conditions and other biotic factors in the two different geographic locations (Table 2). 

**Table 2:** Geographic and climatic characteristics of collection locations.

Collection locations	Sites	Climatic characteristics		
		Sunshine Duration (h)	Mean annual Precipitation (mm)	Mean annual temperature (ºC)
Cangzhou (Sample 1)	Roadsides	2,697	576	11.5
Beijing (Sample 2)	Wheat field	2,780	607	12.5

## Experimental

### Plant material

Flowering aerial parts of *Descurainia sophia* were separately collected in April 2008 from roadsides of the suburb of Cangzhou city, Hebei Province, China, with the geographical coordinate of 38º18´ N and 116º48´ E (S1) and from a wheat field in Liangxiang village, Fangshan district of Beijing city, China, with the geographical coordinates of 39°43′ N and 116°6′ E (S2) and identified by Professor Chaoxian Zhang. A voucher specimen was deposited at Institute of Plant Protection, Chinese Academy of Agricultural Sciences, Beijing, China.

### Isolation of the essential oil

The dried flowering aerial parts were subjected to hydrodistillation for eight hours using a Clevenger-type apparatus, according to the literature [18,19,20]. The sample was added to distilled deionized water (1.5 L) in a 4 L round bottomed flask and heated to boiling, after which the essential oil was evaporated together with water vapour and finally collected in a condenser. The resulting product was then partitioned with ether and dried over anhydrous Na_2_SO_4_. Finally, the solvent was evaporated at reduced pressure. Essential oils were stored frozen at -20 °C until used. The resulting pale yellow oil (30 μL) was solubilized in 1 mL of dichloromethane before the GC injection. 1 μL of this solution was directly used for analysis.

### Gas chromatography (GC)

Gas chromatographic analysis was carried out on a Hewlett-Packard (HP) 5890A Series II gas chromatograph equipped with a split/splitless injector (250 ºC, split ratio 1:30) and a FID operated at250 °C. Chromatographic data were processed with an HP ChemStation 3365-II. An AB-5 fused silica capillary column (25 m × 0.25 mm i.d., 0.25 μm film thickness) was used. The operating conditions were as follows: 5 min at 40 °C initial hold, then from 40-280 °C at 3 °C/min; injector temperature, 250 °C; detector temperature, 290 °C; carrier gas, H_2_ at 1.0 mL/min. Retention indices were determined with C_6_ to C_28 _*n*-alkane standards as reference. 

### Gas chromatography/mass spectrometry (GC/MS)

A Thermo Polaris Q ion-trap mass spectrometer equipped with a Trace GC Ultra gas chromatograph (Waltham, MA, USA) with a split/splitless injector was used for mass spectrometric identification of the GC components and quantitative composition. 1.0 μL of the essential oil solubilized in dichloromethane was injected in splitless mode. A fused-silica capillary column AB-5MS (5% diphenyl-95% dimethylpolysiloxane; 60 m × 0.25 mm i.d., 0.25 μm film thickness) was employed with helium (purity 99.999%) as carrier gas at a constant flow-rate of 1.0 mL/min. The column temperature was programmed as follows: 40 °C for 1 min and directly to 260 °C at 3 °C/min and holding for 10 min. The inlet pressure was 200 kPa, the linear velocity 1.0 mL/min (70 °C). The injector temperature was kept at 250 °C. The temperatures of the ionization source and transfer line were 250 and 280 ºC, respectively. The electron energy was 70 eV. Mass spectra were obtained by automatic scanning of the mass range m/z 40-625 a.m.u. at 2.0 scan/s.

### Identification of components

The individual peaks were identified by comparison of their retention indices to those of authentic samples, as well as by comparing their mass spectra with the NIST 05 library mass spectral database and literature [21,22,23,24,25]. The percentage composition of compounds (relative quantity) in the essential oil was computed from the GC-FID peak areas using the normalization method, without correction factors.

## Conclusions

Analyses of the volatile constituents from the plants of *Descurainia sophia* collected from different growing locations indicate *cis*-β-ocimene, menthol, neoisomenthyl acetate to be the predominant components of *Descurainia sophia*. This work provides the first report of the analysis of essential oils of *Descurainia sophia* from different habitats in China.
